# Non-Symmetrical Direct Extrusion—Analytical Modelling, Numerical Simulation and Experiment

**DOI:** 10.3390/ma14247856

**Published:** 2021-12-18

**Authors:** Marek Kowalik, Piotr Paszta, Tomasz Trzepieciński, Leon Kukiełka

**Affiliations:** 1 Faculty Mechanical Engineering, Kazimierz Pulaski University of Technology and Humanities in Radom, 54 Stasieckiego Street, 26-600 Radom, Poland; 2 Faculty of Mechanical Engineering and Computer Science, Czestochowa University of Technology, al. Armii Krajowej 21, 42-201 Częstochowa, Poland; 3 Faculty of Mechanical Engineering and Aeronautics, Rzeszow University of Technology, al. Powst. Warszawy 8, 35-959 Rzeszów, Poland; tomtrz@prz.edu.pl; 4 Department of Mechanical Engineering, Koszalin University of Technology, 15-17 Racławicka Street, 75-620 Koszalin, Poland; leon.kukielka@tu.koszalin.pl

**Keywords:** co-extrusion, FEM, hollow profiles, non-symmetric extrusion, numerical modelling

## Abstract

The article presents the original technology of the extrusion of hollow curved pipes. The curvature radius of pipe axis was obtained directly during extrusion by eccentric alignment of the annular calibration gap of the extrusion die. Theoretical relationships describing the radius of curvature of the extruded part as a function of the eccentricity e of position of the annular calibration gap in the die were developed. A die with replaceable inserts with eccentricity e equal to 1, 2, 3, 5, 7 mm was designed and fabricated. Experimental tests were carried out to extrude lead pipes with an outer diameter of 20 mm and an inner diameter of 18 mm. Measurements of the radii of the curvature of the extruded pipes were consistent with the values calculated from the developed theoretical relationships. Numerical modelling of the proposed method of extrusion in a finite element-based QForm 3D program was carried out. The finite element method (FEM) numerical calculations were carried out for lead. Numerical simulations and experimental studies have shown that, by changing the value of the eccentric gap, the radius of curvature of the extruded pipe can be controlled.

## 1. Introduction

One of the methods used in the production technology of machine parts, including the production of hollow toric elements, is the direct extrusion of metal elements. Extrusion is a process used to create objects of a fixed cross-sectional profile. During the extrusion process, the material is placed in the die and is subjected to the pressure exerted by the punch. The extruded material flows out through the die opening or the gap between the punch and die. The cross-section of the extruded stock is reduced and the length increases. The state of stress in the major part of the plasticized material is a triaxial nonuniform compression [1,2,3,4].

Extrusion is one of the most modern methods of plastic processing of nonferrous metals. Extruded parts do not require additional finishing, and it is possible to obtain products with very accurate dimensions. In extrusion, higher degrees of plastic deformation are obtained than with other methods of plastic working. Large plastic deformations in this technology require the use of a large extrusion force [5]. The extrusion process is usually limited by the strength of the die [6]. The advantage of the extrusion process compared to other production processes is the possibility of obtaining products with complex cross-sections. The appropriate selection of technological parameters should ensure the high accuracy of the extruded elements.

Research on the problem of the extrusion of asymmetrical elements in the cross-section and with straight axes are focused on the use of the extended calibrating part of the matrix [7]. Asymmetrical extruded profiles have a tendency to warp. In order to reduce this warping, frictional resistance is increased by using an increased length of the calibrating part in the die [8].

An analysis of the literature on the recommended sizes and accuracy of rods and pipes manufactured by the extrusion method allows one to conclude that the extruded parts do not have a straight axis, however their axis has a very large radii of curvature [9]. In the scientific literature on extrusion, there is little data on the extrusion of asymmetrical and intentionally curved elements. The previous theoretical analyses for solving this problem for complex profiles have been conducted mainly in terms of the calculation of extrusion force, work, as well as the deformations necessary for the realization of this process [10].

The pioneering analytical work on the extrusion of bars with various types of asymmetric cross-sections has been carried out by Napgal and Altan [11]. They determined the analytical function of creating a stream of metal necessary to calculate the extrusion force of elliptical bars. Basily and Sansome [12] proposed the upper bound theory (UBT) for the problem of drawing square bars from a round stock, while Yang and Lee [13] kinematically determined the permissible velocity fields when extruding bars with generalized cross-sections, i.e., when the cross-section similarity was maintained throughout the whole period of deformation. Prakash and Khan [14], following the principle of geometric similarity, presented a solution for the process of extrusion and the drawing of polygonal profiles using the upper-bound solution under the assumption of a straight stream of metal flow. Boer et al. [15] described the method of upper-bound solution of the process of drawing square bars from a round stock using coordinate transformation. Gunasekera et al. [16] conducted an investigation on the process modelling of hot forging and the extrusion of aircraft structural parts. They also developed an efficient software for the analysis of metal flow during the forging of complex shapes. Kiuchi et al. [17] developed an analytical method in order to investigate the extrusion and the drawing processes of various kinds of non-symmetric cross-sections. It was possible to predict the optimal shape and dimension of the die for the required product.

Research on modelling asymmetric plastic flows was also carried out by Piwnik [18]. He proposed plastic flow patterns enabling the calculation of the extrusion force of a cylindrical bar through a die with an eccentric circular hole, taking into account the geometric and material factors influencing the extrusion force. Problems related to forces in plastic forming processes are concerned with determination of the pressures and forces between the tool and the material formed [19], as well as the friction conditions [20]. Abrinia and Ghorbani [21] presented a new generalized formulation for the analysis of the forward extrusion of non-symmetric sections.

Chitkara and Celik [22,23,24] proposed an analytical method based on the UBT to investigate the three-dimensional, off-centric extrusion of arbitrarily shaped sections from arbitrarily shaped billets through linearly converging and smooth curved dies. For a given reduction in an area, curvature of the extruded product as well as the extrusion pressures may be obtained. Abrinia et al. [25] proposed a new formulation of a kinematically admissible velocity field based on a variable axial velocity component. Libura and Rękas [26] showed that the extrusion process is characterized by the very high deformation degree e. Metal flow may be predicted through finite element-based numerical models. They also described the procedure of designing dies for the extrusion process of complex shapes from aluminium alloys. Qamar [27] presented the fact that, non-symmetric dies, which are quite common in commercial extrusion practice, would have an even more inhomogeneous metal flow. It was found that the asymmetrical geometry about the longitudinal axis resulted in extrusion defects known as twists or bends.

Zhou et al. [28] presented a novel energy-efficient forming method based on differential velocity sideways extrusion (DVSE), which can directly form billets into curved profiles/sections by extrusion. A cold deformation of round bars and tubes was examined. It was found that curved profiles/sections with no distortion can be formed. Yu et al. [29] and Zhou et al. [30] proposed a new extrusion technology, in which two punches are used. An application of the technology developed to form lightweight, curved structural profiles was proposed. The technology is known as DVSE, and includes welding extrusion, hot extrusion, and co-extrusion.

Chen et al. [31] studied the effect of the process parameters on the shape of the extruded tube in the multi-hole extrusion of aluminium alloy tubes by both FE-based analysis and experimental methods. Min et al. [32] used the Arbitrary Lagrangian–Eulerian method to simulate the natural bending deformation of the sheet metal. A self-bending extrusion die for a workpiece was designed via a spline curve, with an inclination as the contour of the deflector chamber. Takahashi et al. [33] investigated the effects of die dimensions on the curvature of extruded rectangular bars by experiments and FE modelling.

Pb1 lead is the basic model material used in the analysis of plastic processing [34] due to its excellent formability. Pipe bends (U-bends) are used for the construction of pipelines transporting liquid and gases in electric power stations and refineries. They are also used in any buildings, to which water and sewage and heating systems are connected. The method of extrusion of curved tubular elements can be applied to all materials that can be plastically processed, in particular metals, e.g., steel, copper, aluminium and its alloys. It is also possible to manufacture parts from thermoplastic materials that are conventionally processed using extruders such as polyvinyl chloride, polyamide, polypropylene, and polyethylene. However, lead mouldings can be used to build chemical equipment that is exposed to sulfuric acid and hydrochloric acid due to its anticorrosive and nonreactive properties.

A comprehensive approach to the problems of widely used curved profiles is taken in the paper [35]. In this paper, the processes for innovative development in the field of forming curved profiles are identified. The non-symmetrical direct extrusion, which can directly form the billets into curved profiles by one single extrusion, is proposed. The direct extrusion method, which was proposed in this study, makes it possible to obtain a bent thin-walled closed profile with a specific bending radius directly from the extrusion process without an additional bending operation. The control of the bend radius of the extruded thin-walled profile is achieved through the use of an appropriate die design with an eccentric shift of the calibration hole.

## 2. Theoretical Analysis

### 2.1. Eccentric Direct Extrusion Process

**Figure 1:**
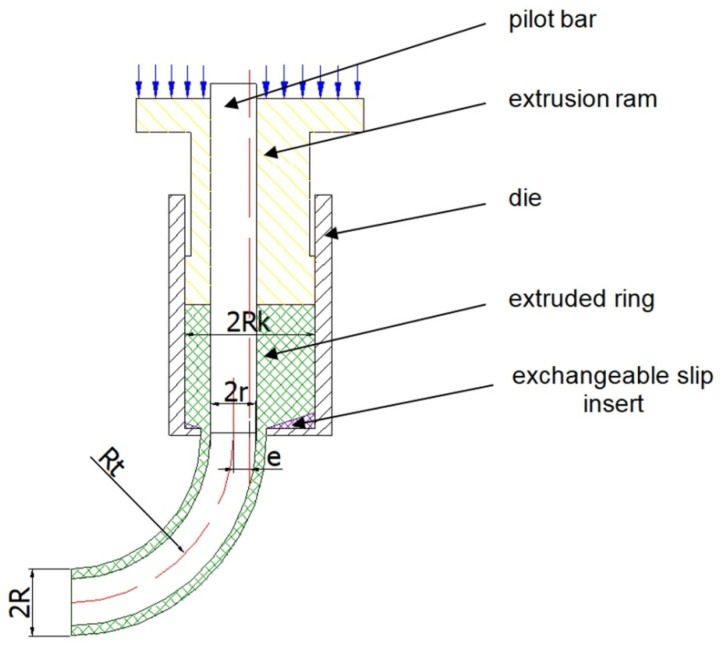
Scheme of method radial formation of a hollow torus piece.

A general schematic diagram of the formation of hollow torus pieces by the direct extrusion method is shown in Figure 1. The method of the direct extrusion of hollow parts with a nonrectilinear profile radius consists in the use of the eccentric positioning of the hole in the die.

Due to the eccentric position of the hole in the die bottom, on both sides of the plane of symmetry of the wall-forming gap, a difference in material volumes occurs, while the different material volumes flowing into the ring-shaped gap causes the extruded piece to bend. A special type of equipment was designed and constructed for tests. A scheme of equipment, together with a direct-extruded piece, is shown in Figure 1.

The die hole includes blank centring and forming parts. The working edge of the die hole should be inclined to the axis at the angle 2Θ ranging from 30 to 180°. It has been assumed that, when the ram moves along the axis of symmetry of the die container, the unit volume of the material extruded by the ram in the vertical direction is identical at any point of the ram active cross-section. This vertical displacement causes the material streams to flow over the die bottom towards the ring-shaped gap, limited by the inner radius r of the extruded piece and the outer radius R of the extruded piece. In the ring-shaped gap, they change the direction of flow to vertical. By flowing into this gap, the material forms the wall of the hollow circular ring piece. This description represents a qualitative formulation, while the quantitative formulation requires geometrical analysis, which is presented below.

### 2.2. Formation of the Hollow Torus Profile

**Figure 2:**
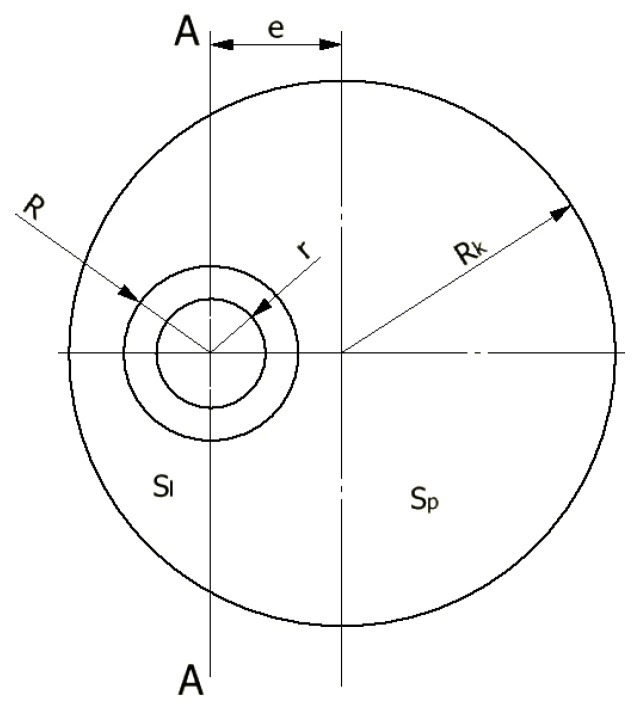
Dimensions of extrusion die: e—eccentric; R_k_—radius of stock; R—outer radius of extruded piece; r—inner radius of extruded piece; S_l_—stock surface to the left of the AA axis; S_p_—stock surface to the right of the AA axis.

On the basis of the presented description, we can assume that the volume of material forming the left and right sides of the axis AA of the hollow torus are different and depend on the eccentric value of e (Figure 2).

With the constant height of the initial ring, these volumes are directly proportional to the areas: S_l_ to the left and S_p_ to the right of the axis AA passing through the centre of the ring hole. Assuming the incompressibility of the material, and neglecting any internal friction phenomena, the geometrical analysis of the torus pieces formed can be performed. With the assumptions and the division of surface areas into S_l_ and S_p_, the radius R_t_ of the extruded piece is defined by the function of four independent variables in the form of R_t_ = f(r, R, R_k_, e), which is derived below.

**Figure 3:**
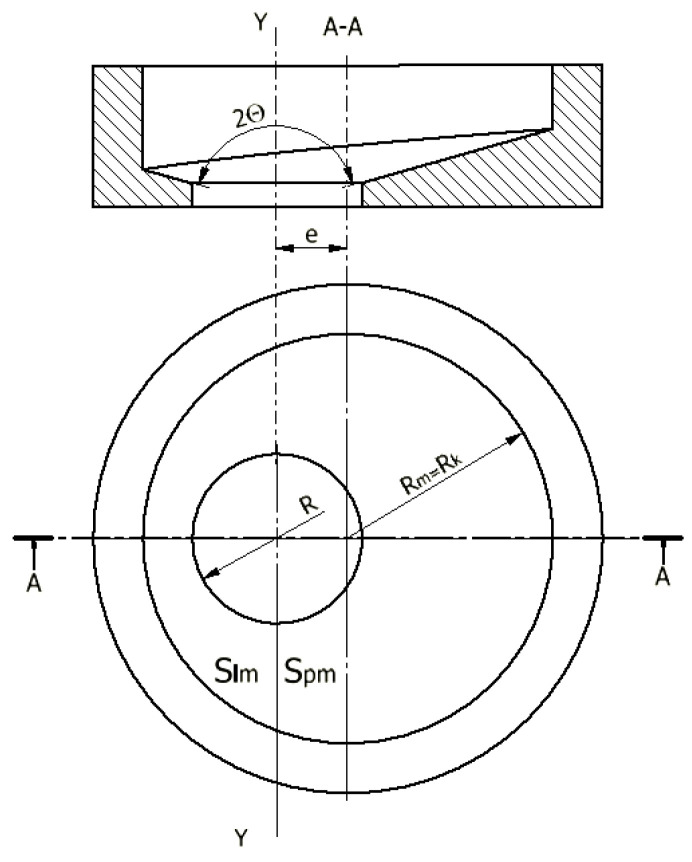
Shape of the die bottom area for the variable angle Θ of material flow into the flow gap: e—eccentric of the die calibration hole; R—radius of the die calibration hole; R_m_ = R_k_—die radius; S_lm_—the surface of the die bottom to the left of the YY axis; S_pm_—the surface of the die bottom to the right of the YY axis; Θ—inclination angle of the die bottom.

Then, we determine from Figure 3 that the die bottom surface area, *S_lm_*, to the left of the plane of the intersection along the axis Y is:

(1) $$S_{lm}=\sqrt{1+ctg^{2}\Theta }\left[\frac{\left(R_{k}^{2}-R^{2}\right)\pi }{2}-e\sqrt{R_{k}^{2}-e^{2}}-R_{k}^{2}\arcsin\frac{e}{R_{k}}\right]$$

Whereas, the die bottom surface area, *S_pm_*, to the right of the intersection plane is:

(2) $$S_{pm}=\sqrt{1+ctg^{2}\Theta }\left[\frac{\left(R_{k}^{2}-R^{2}\right)\pi }{2}+e\sqrt{R_{k}^{2}-e^{2}}+R_{k}^{2}\arcsin\frac{e}{R_{k}}\right]$$

Ultimately, the total surface area of the conical die bottom of the variable angle Θ is:

(3) $$S_{m}=S_{pm}+S_{lm}=\pi \sqrt{1+ctg^{2}\Theta }\left(R_{k}^{2}-R^{2}\right)$$

### 2.3. Volume of Material Flowing within the Gap

**Figure 4:**
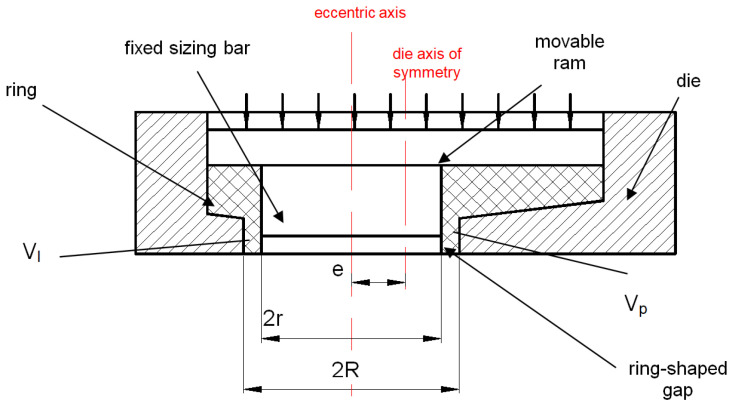
Forming of the torus end with the division of volumes into V_l_ and V_p_ in relation to the eccentric axis: e—eccentric; r—hollow radius of extruded piece; R—outer radius of extruded piece.

With the uniform flow of material into the ring-shaped gap (Figure 4), the ratio of the volume *V_l_* of the formed torus to the left and the volume *V_p_* to the right of the torus axis is proportional to the corresponding areas:

(4) $$\frac{V_{l}}{V_{p}}=\frac{S_{l}}{S_{p}}$$

**Figure 5:**
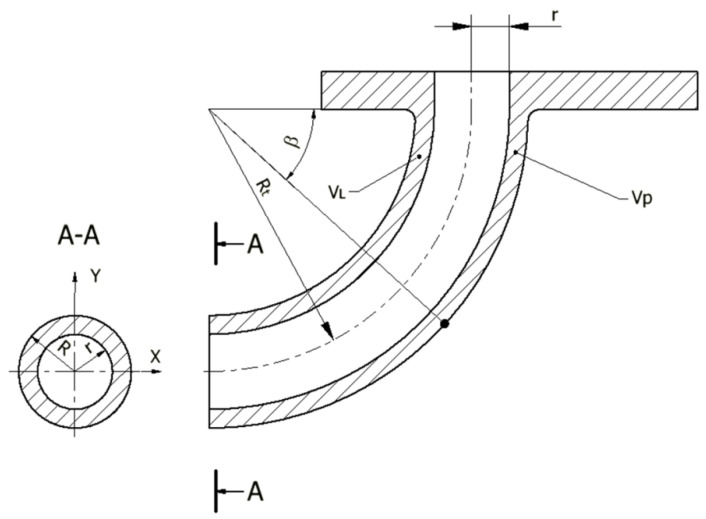
Shape and dimensions of the torus end: R_t_—radius of extruded piece; V_L_—the volume of the hollow torus to the left of the cross-section; V_P_—the volume of the hollow torus to the right of the cross-section; R—outer radius of extruded part; r—inner radius of extruded piece; β—the angle of curvature of the part.

The volumes V_l_ and V_p_ of the hollow torus of the fixed radii r, R has an effect on the values of the radius R_t_ of the formed torus and the values of the angle β of the elbow curvature (Figure 5).

The formed torus end is composed of a flange and the hollow torus with the radii r, R, whose volume depends on the angle β and radius R_t_. Thus, the volumes of the left, V_l_, and the right, V_p_, sides of the torus are defined by the functions of the following quantities: r, R, R_t_, β. These functions are determined by Guldin’s rules, according to which the volume of the solid formed by the rotation of the specific surface S by the angle *β* about the axis *x* is defined by the following relationship:

(5) $$V=\beta \int_{S}xdxdy$$

The volume *V_l_* of the hollow torus has the form of:

(6) $$V_{l}=\frac{\pi }{2}\beta R_{t}\left(R^{2}-r^{2}\right)-\frac{2}{3}\beta \left(R^{3}-r^{3}\right)$$

The volume *V_p_* of the hollow torus has the form of:

(7) $$V_{p}=\frac{\pi }{2}\beta R_{t}\left(R^{2}-r^{2}\right)+\frac{2}{3}\beta \left(R^{3}-r^{3}\right)$$

Given the volumes of the hollow torus calculated from Equations (6) and (7), the torus radius *R_t_* has been determined:

(8) $$R_{t}=\frac{2\left(R^{2}+Rr+r^{2}\right)\left(R_{k}^{2}-r^{2}\right)}{3\left(R+r\right)\left(R_{k}^{2}\arcsin\frac{e}{R_{k}}+e\sqrt{R_{k}^{2}-e^{2}}\right)}$$

Equation (8) can be simplified using known transformations in the case where *e* << *R*. We obtain a simplified formula for calculating the radius *R_t_* of the torus for practical industrial extrusion applications:

(9) $$R_{t}=\frac{\left(R^{2}+Rr+r^{2}\right)\left(R_{k}^{2}-r^{2}\right)}{3eR_{k}\left(R+r\right)}$$

The radius of the torus *R_t_* depends on the outer *R* and inner *r* radiuses of the workpiece, the radius of the die *R_k_*, and the eccentricity of the die *e*. Equation (9) shows that the value of the radius *R_t_* of the toric pipe, formed by using the direct extrusion from a stock with dimensions *R_k_*, *r*, *e*, and *R,* is inversely proportional to the value of the eccentricity *e*.

## 3. Experimental Apparatus and Methods

**Figure 6:**
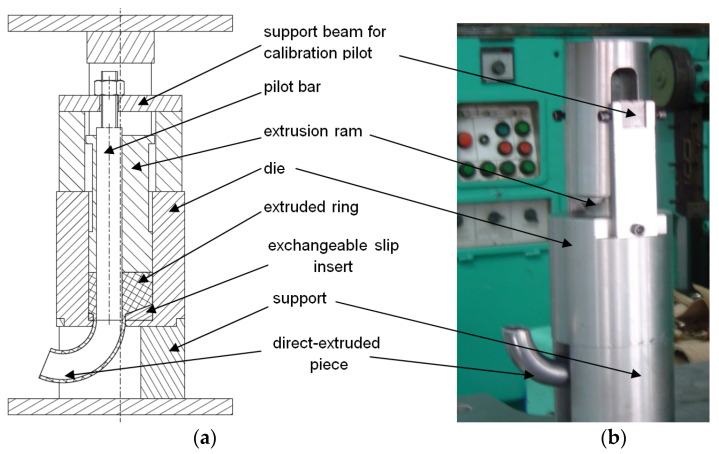
(**a**) schematic diagram of test stand for toric pipe extrusion and (**b**) view of the extrusion die with the extruded piece.

Experimental studies of direct extrusion of pipes were carried out using an eccentric die (Figure 6) mounted on a PYE 250-N hydraulic press (Wema Zeulenroda Erfurt). The main parts of the test fixture were: die with eccentric hole, punch, mandrel calibrating the hole in the extruded part, base of the fixture, brackets, beam fixing the brackets. Other parts for clamping and fixing the jig, such as pins, gauges, screws, nuts, were also used. All of these parts were assembled into a jig, which was then set up on a press.

The die was made of 145Cr6 steel in the form of a rolled bar and softened when annealed. The die hardness was in the range 60–62 HRC. A charge material with a diameter of 40 and a height of 25 mm was used as the workpiece. Five dies with 18 mm diameter holes positioned eccentrically (e = 1, 2, 3, 5, and 7 mm) were used in the experiments. The hole in the bottom of the die for extruding torus parts had a cylindrical part and a working part for shaping the moulded part. Eccentric displacement values were assumed to be in the range of 1–7 mm for a cylindrical hole with a diameter of 40 mm. A calibration ring height of 4 mm was adopted to reduce friction and unit pressure transferred by the tool. The dimensions of the punch were adjusted by design to work with the die as a motion joint. The tolerance of the punch diameters and the die receptacle openings were selected so that no circumferential discharge would occur during extrusion, but, at the same time, to ensure free entry of the punch into the die. A sliding fit of the punch in the die was used.

The punches were made from X165CrV12 cold-work tool steel, resistant to abrasion. In the punch, the working part is a flat face. This shape of the surface reduces the waste of material remaining after extrusion in the die edge zone.

The required concentricity of the jig was achieved by guiding the punch in the die bore as well as on a part of the calibrating mandrel. The design of the punch is different to those commonly used, as it had a hole eccentrically offset to guide the calibrating pin. In this way, the preassembled jig with a suitably sized sample was placed on the press table. It was then loaded by applying pressure to the punch. The shape and dimensions of the jigs were designed depending on the initially assumed sizes of the hollow torus elements for the specified extrusion method and the assumed technological parameters of the process.

For all cases under analysis, forming on a direct-supplied hydraulic press of a pressure force of 50 MN and an adopted speed of pressing (movement of stamp) was 0.4 mm/s.

Technological tests carried out have shown that a uniform material flow in the die is obtained in a wide range of punch travel speeds. Moreover, uniform wall thickness is obtained along the entire torus length. Lead was used as the material for preliminary studies. Preliminary tests and their analysis showed that no cracks or scratches were observed on the external surfaces of the torus elements and that the surface roughness both outside and inside the element was satisfactory. From the tests and observations made, it can be concluded that the proposed method of corotational extrusion shows a clear technological advantage over the methods used so far to produce hollow torus-shaped elements. Manufactured devices for extruding hollow parts with torus outlines according to the proposed design have a satisfactory strength to load, necessary stiffness, and resistance to wear. The correctness of their design has been verified by tests.

A series of tests for the direct extrusion of Pb1 lead hollow elements with the strength parameters given in Table 1 were carried out. The contours of the obtained elements were measured. The results of the measurements are presented in Table 2 and Table 3.

## 4. Experimental Results

### 4.1. Extrusion Process

Table 2 shows the values of the radius R_t_ of the torus with an outer diameter of 20 mm and an inner diameter of 18 mm, obtained in experimental tests of forming the extruded pieces from a stock with a diameter of 40 mm. The data in Table 2 were compared with the values calculated on the basis of the theoretical formula [9].

Table 3 shows the values of the torus radius R_t_ of torus with an outer diameter of 22 mm and an inner diameter of 18 mm obtained in the experimental tests on the forming of the extruded pieces from a stock with a diameter of 40 mm. The data in Table 3 were compared with the values calculated on the basis of the theoretical formula [9].

**Figure 7:**
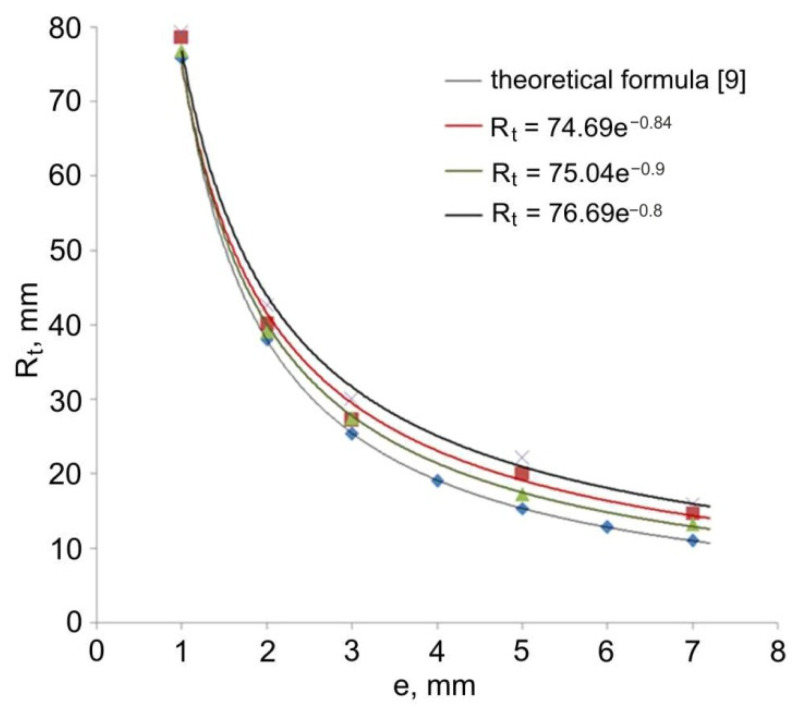
Comparison of the theoretical and experimental values of torus radius R_t_ (outer radius of workpiece 2R = 20, inner radius of workpiece 2r = 18 mm, diameter of stock 2R_k_ = 40 mm).

**Figure 8:**
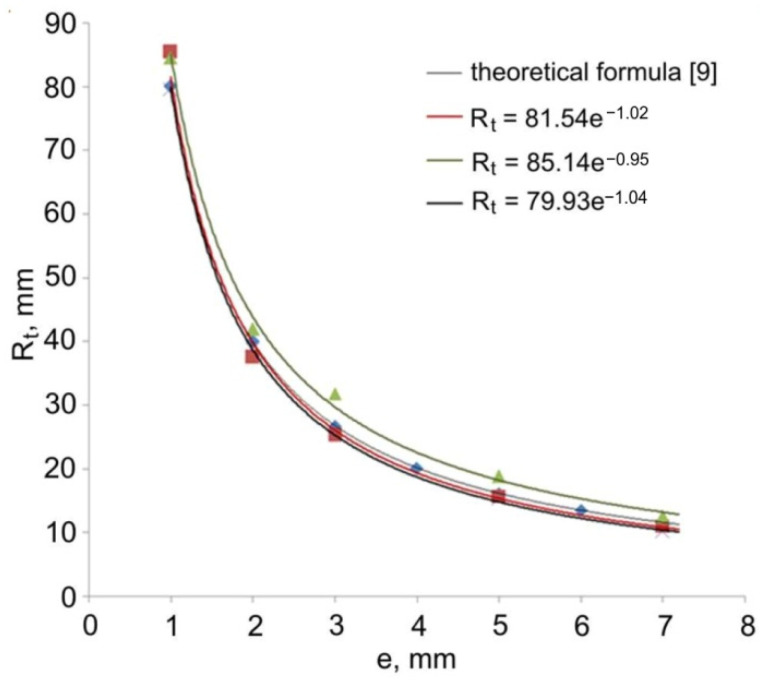
Comparison of the theoretical and experimental values of torus radius R_t_ (outer radius of workpiece 2R = 22, inner radius of workpiece 2r = 18 mm, diameter of stock 2R_k_ = 40 mm).

Trend lines shown in Figure 7 and Figure 8 were determined based on the regression analysis. The regression functions for all experimental trials have the nature of an exponential function R_t_ = f(e^x^). The value of the radius R_t_ of the hollow elements formed by the direct extrusion method from a specific stock is inversely proportional to the value of eccentric e. The presented theoretical and measured values are convergent. The observed shift of the theoretical plot in relation to the experimental results by a constant value may be caused by the influence of technological parameters of the extrusion process, in particular the axial position of the calibrating pin in relation to the die face, friction conditions, and elastic deformation of tool during the forming process.

### 4.2. Microstructural Examination

**Figure 9:**
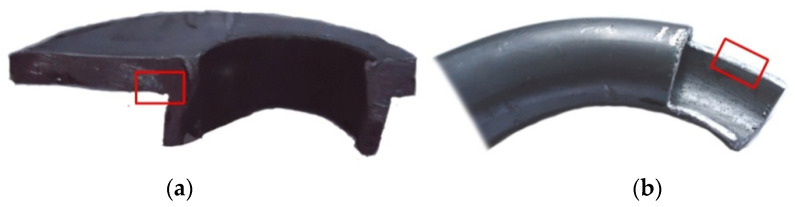
Sampling areas for metallographic tests: (**a**) flange part of the molding, (**b**) the torus molding.

The microstructure of the specimens was examined using the Digital Microscope KEYENCE VHX-7000 using two lenses, the Z20 with a magnification of ×20–200 and the VH-Z100UR with a magnification of ×100–1000. The tests were carried out on samples collected in the area of the die gap (Figure 9a) and the torus part of the workpiece (Figure 9b). The specimens were etched with the use of CH_3_COOH + 30% H_2_O_2_ solution.

**Figure 10:**
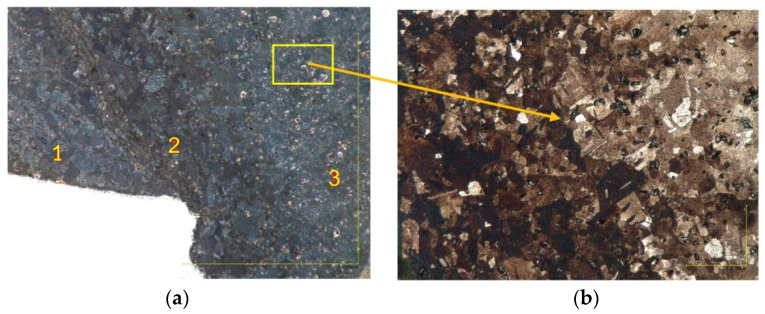
Microstructure of the specimen in the flange part of molding made from lead stock: (**a**) material flow zone (×50 magnification) and (**b**) the same area with ×500 magnification.

Lead has a recrystallization temperature below room temperature [36,37]. Therefore, it is difficult to observe deformed microstructure. Thus, the same moldings were made of lead balls with a diameter of 2 mm in order to more conveniently observe deformations and material flow in the molding. Figure 10 shows the microstructure of the material in the flange part of the molding (Figure 9a) at a 50× magnification. Zone 1 denotes the dead zone, in which the intensity of the material flow is the lowest, zone 2 denotes the area where intense material flow takes place in the radial and axial directions with intensive mixing of the material, and zone 3 denotes the area where the axial flow of material dominates.

**Figure 11:**
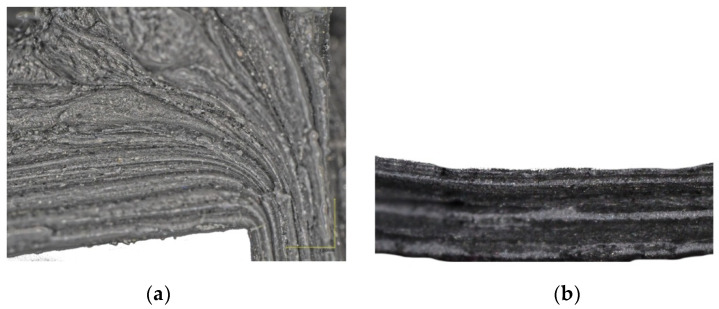
The fracture of the flange part and the wall of the molded part made from lead balls: (**a**) micrograph of the area of the die edge (×50 magnification), (**b**) torus wall (×50 magnification).

Figure 10b shows equiaxial lead α grains of various sizes. The microscopic image shows the occurrence of recrystallisation processes and further grain growth, as evidenced by large differences in grain sizes. Figure 11a shows the flange part of molding with a characteristic change in the direction of the metal flow. Darker bands were formed from the oxidized surface of the shot, while the lighter areas are unoxidized material. Figure 11b presents the wall of the molding. This surface was formed after breaking the molding along the lead shot flow direction. Black strands of oxidized lead and gray bands of unoxidized lead are visible and are arranged parallel to each other. Microscopic examinations showed that, in the tested eccentric extrusion process, the material flow is laminar. There are no material cracks or other material defects caused by the different flow rate of the material through the die gap.

## 5. Numerical Modelling of Manufacture of Hollow Torus Pieces

In order to develop a new technological process, it is advisable to verify its course in the best possible way and in the shortest possible time, and to select the technological parameters of the process itself and the equipment. Assuring such conditions is possible by a method of numerical computations using the QForm 3D environment. The numerical computations enable subsequent experimental tests to be properly oriented.

The development of tooling to obtain hollow parts with the appropriate radius by experimentation may fail to bring the expected results, requires a lot of experience in their design, and is quite expensive. In order to develop a new technological process of extrusion, it is advisable to verify its course in the best possible way, in the shortest possible time, and to select the technological parameters of the process itself and of the tooling. Such conditions can be created by numerical calculations, as they make it possible to properly focus the experimental research later on.

The simulation allows for the accurate prediction of material flow, analysis of die filling, possible deformations, and the defects of the workpiece caused by flow. There are studies of the extrusion process for the profiles of angles and pipes and also of extrusion through multi-hole dies, in which the bending of the moulded part with respect to the extrusion axis is achieved. In these studies, the aim is to achieve a uniform metal flow and to obtain a straight axis of the extruded part [38,39,40,41].

Thanks to the application of numerical modelling in the spatial state of deformation, it is possible to implement extruded components in dies with an eccentrically placed calibration slot. The corotating extrusion process of a hollow torus component has been analysed, and the measurements was taken.

The following boundary conditions were adopted for computations using the QForm 3D program: an initial tool and charge of 20 °C, and a coefficient of friction between the tools and the deformed change of 0.2–0.5, which corresponds to the real conditions with conventional lubrication applied.

The extrusion process was conducted in a die with a container diameter of 40 mm, a sizing bar diameter of 18 mm, and a die sizing hole diameter of 20 mm. Pure Pb1 lead was used as the deformed material. The relationship of the yield stress versus temperature and deformation velocity was approximated with Equation (10):

σ = 138.2ε^3^ − 215.3ε^2^ + 117.9ε + 4.694 (10)

Fully automatic finite element mesh generation was used in the QForm 3D program. The self-controlling algorithm ensures an optimal mesh density distribution and that smaller elements are automatically generated in critical areas of the analysis, such as, for example, fillings, areas with more complex details, etc., which ensures accurate results and an appropriate simulation run.

**Figure 12:**
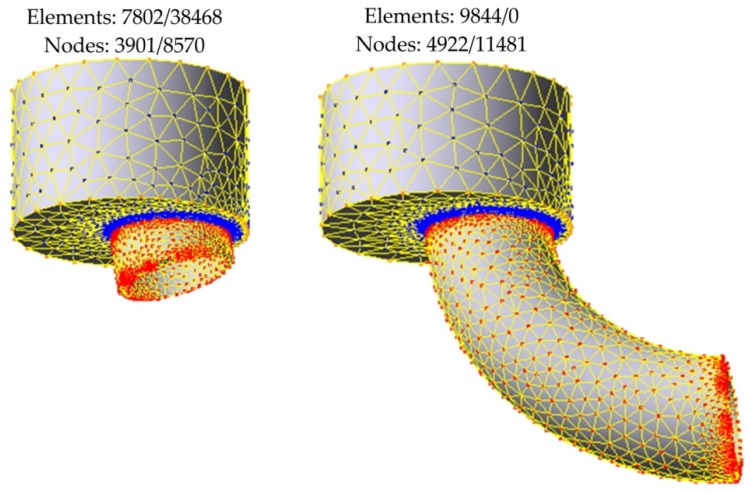
FE mesh of extruded pieces in the following stages of extrusion.

The tool and charge shapes were developed using Autodesk Inventor and were then transferred to the finite, element-based QForm 3D program, where the assumed simulation systems were configured from them. In the next step, a mesh of elements was generated, as the automatic mesh reconstruction was applied, the number of elements changed in each simulation step. Figure 12 shows the finite element (FE) mesh in a workpiece during direct extrusion with the eccentric e = 7 mm.

Too small a size of finite elements may cause problems with solution convergence and ensure the high accuracy of the calculations. This is especially important in the die area, where the material is subjected to high plastic deformation. In the numerical model, the tetrahedral elements were used to discretise the domain analysed. The Qform 3D program allows for automatic mesh refinement to reduce mesh distortion during the analysis [41].

**Figure 13:**
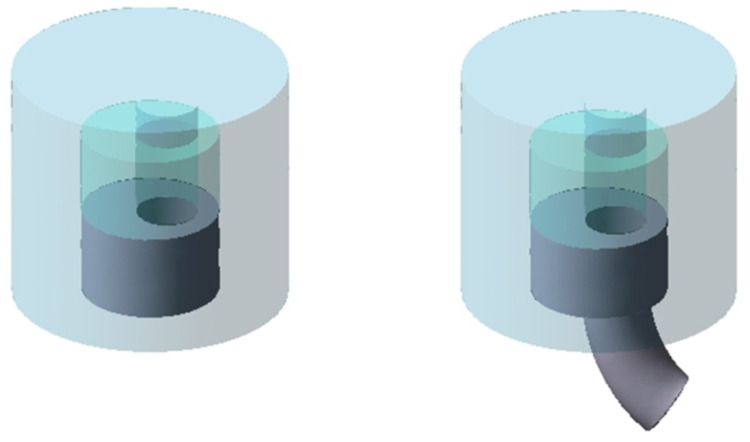

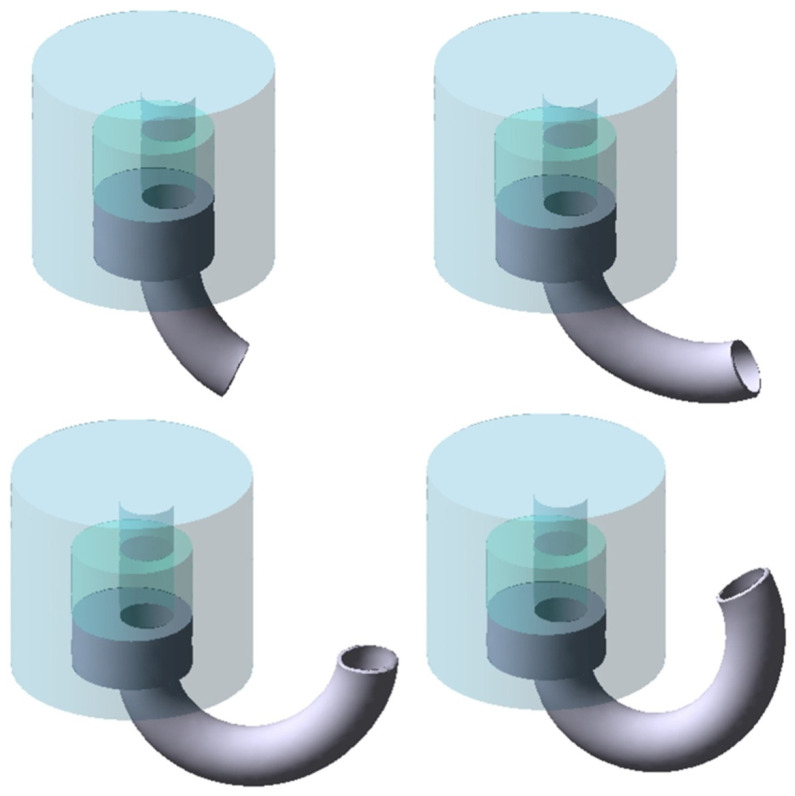
Example shape of a lead torus for 5 mm eccentric displacement.

The numerical analysis performed included cases of concurrent extrusion, in which the eccentric offset of the calibrating part was changed. The process was carried out in a spatial state of strain. An example of an extruded piece shape for an eccentricity of 5 mm is shown in Figure 13.

## 6. Numerical Results and Discussion

### 6.1. Analysis of the Velocity and Flow Lines of the Lead Charge

**Figure 14:**
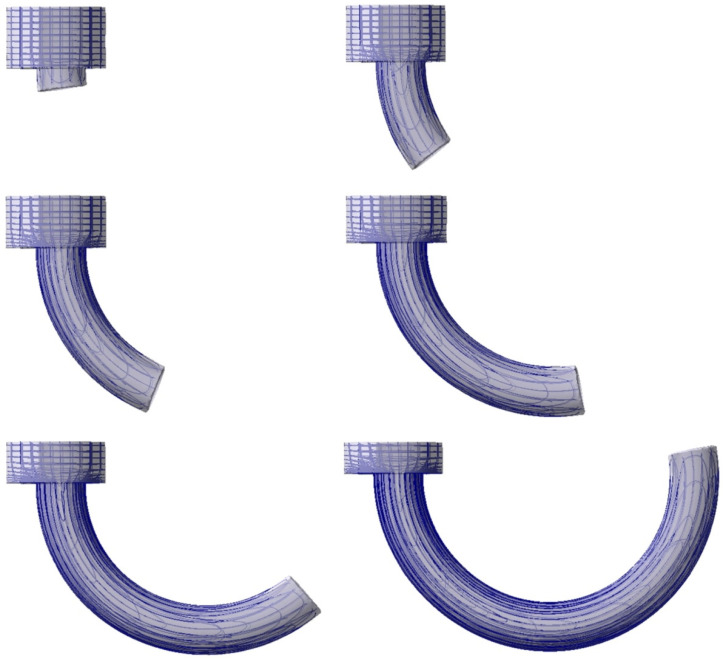
Lines of flow of lead in the successive phases of the extrusion process, visible on the deformed grid in the longitudinal section.

The three-dimensional graphical effects of the simulation are presented instantaneously during the course of the simulation. Extrusion pieces of an internal diameter of 20 mm and a hollow diameter of 18 mm were obtained. Characteristic computation results were presented in the form of coloured maps and diagrams. Figure 14 shows an example of the numerical simulation of the lines of the flow of lead during direct extrusion with an eccentric position of the sizing ring.

It was observed that the strain grid allowed a very accurate reproduction of the course of metal plastic deformations. It has been found that, by using eccentric extrusion, hollow products are obtained, which are distinguished by a uniform fibrous structure composed of elongated crystallites in the direction of flow.

On the die bottom, elastic areas form where the material does not deform plastically; these are so-called dead zones. A dead zone has the shape of a truncated hollow cone of the revolution of an angle ranging from 106° to 108°, lying in the eccentrically positioned axis of the die hole, oriented with its vertex in the material flow direction, and limited with the cylindrical die surface from the outside.

### 6.2. Analysis of the State of Stress

**Figure 15:**
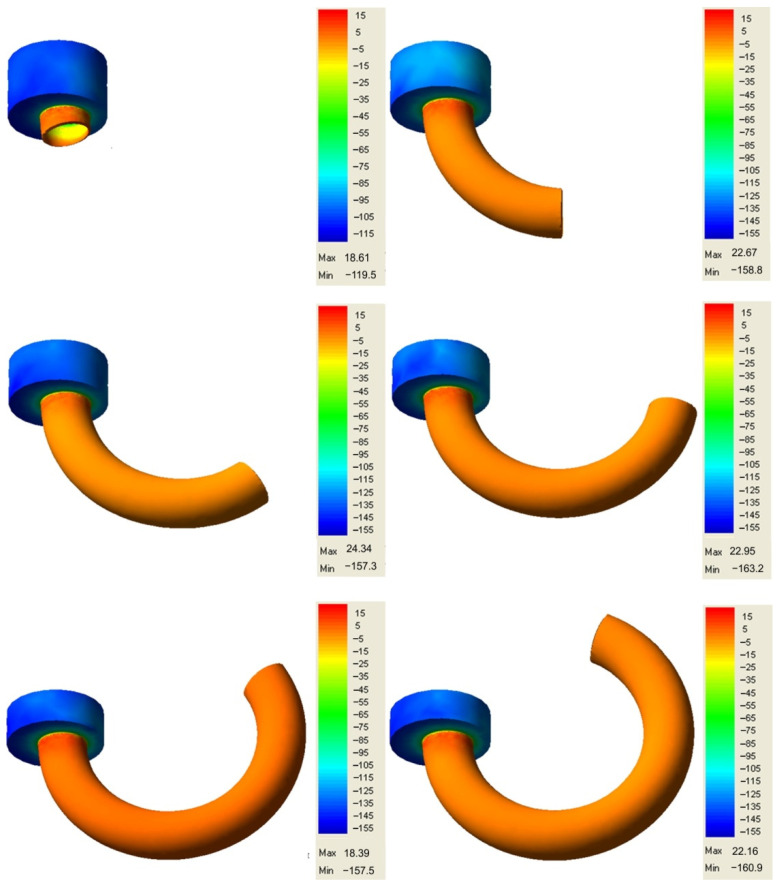
Mean stress distribution (in MPa) during eccentric extrusion (e = 3 mm) of lead.

The distribution of stresses during the process of extruding the hollow torus is complex in character. During the extrusion process, the lead charge is deformed and is subject to the ram pressure force, friction forces, and forces associated with die and sizing bar reactions. Part of these forces deform the charge, and the other part attempts to block the movement of the metal particle relative to each other and in relation to the die and bar surfaces. Under the effect of the above-mentioned active and reactive forces, the material remains in a state of stress. In the analysed extrusion process, triaxial compression occurs in the plastic deformation region, owing to which the metal has the best plasticity in these conditions. A stress pattern exists here, which is the most advantageous in terms of plasticity, and which includes one tensile strain and two compressive strains. Conditions occur here, in which the lead under compressive stresses flows in the direction of the highest stress gradient from the surface of contact of the ram face with the metal, on which the mean stresses reach the highest magnitude, to the ring-shaped die hole, where, on the surface of outflowing lead, the normal stresses attain a zero value. The distribution of mean stresses is illustrated in Figure 15.

During the extrusion of a lead ring through the die ring-shaped hole, compressive stresses occur in the entire volume of the material being deformed, which vary in the extrusion direction in a continuous manner from about −160 MPa to 22 MPa. The value of 22 MPa can be explained by the effect of a large diameter reduction during extrusion, the occurrence of a dead zone, a particular effect of metal friction forces in the sizing gap, and, to a small extent, by an additional displacement of metal particles in the transverse direction, as the equalization of particle movement velocities may give rise to tensile stresses. The mean stress on the external surface amounts to approx. −150 MPa and is higher compared with the stress forming in the zone of contact of lead with the sizing bar.

The presented distribution of stresses can also be related to the changes in temperature within the die. In the deformation region within the die around the ram, the deformation resistance decreases and the average stress ranges from −15 to −55 MPa, whereas, in the areas near the die surface, the deformation resistance is higher and, at the same time, the stress amounts to approx. −115 MPa. Due to the slow strain rate, the temperature of the tool and workpiece was not analysed. However, the change in temperature depends on the speed of pressing and the duration of the process without cooling the tool [42]. In the free-flow area, the average stress takes on a negative value, which is advantageous in view of the possibility of closing possible material defects. Maintaining the zero value of the mean stress is fully sufficient for attaining plastic flow stability.

**Figure 16:**
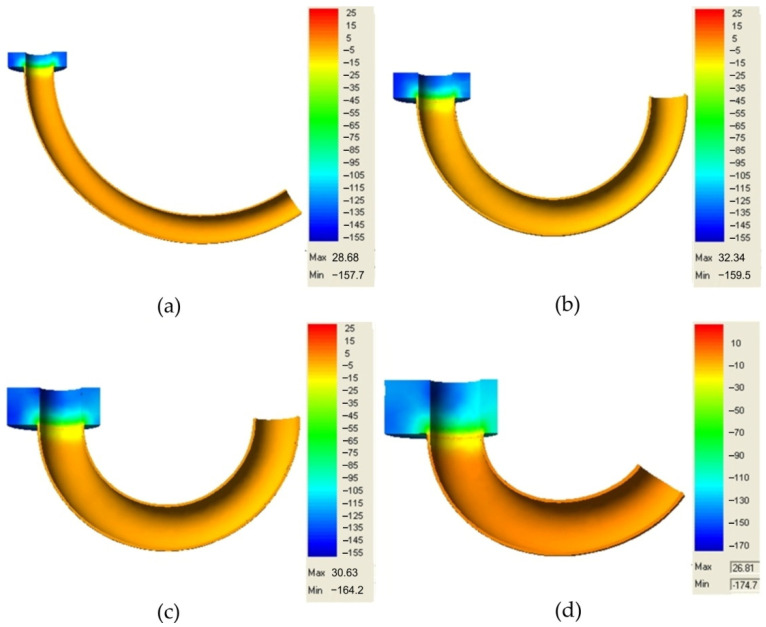
Mean stresses (in MPa) in lead torus, eccentrics: (**a**) 1 mm, (**b**) 2 mm, (**c**) 3 mm, and (**d**) 5 mm.

In the considered cases, as shown in the sections of internal stress map (Figure 16), it is important to know the stresses in the finished extruded piece in order to assess its suitability for proper use.

Based on the results of the extrusion process simulation using the QForm 3D program, a high usefulness of the obtained products can be found. A uniform stress distribution occurs within the extruded piece walls, which is close to zero.

**Figure 17:**
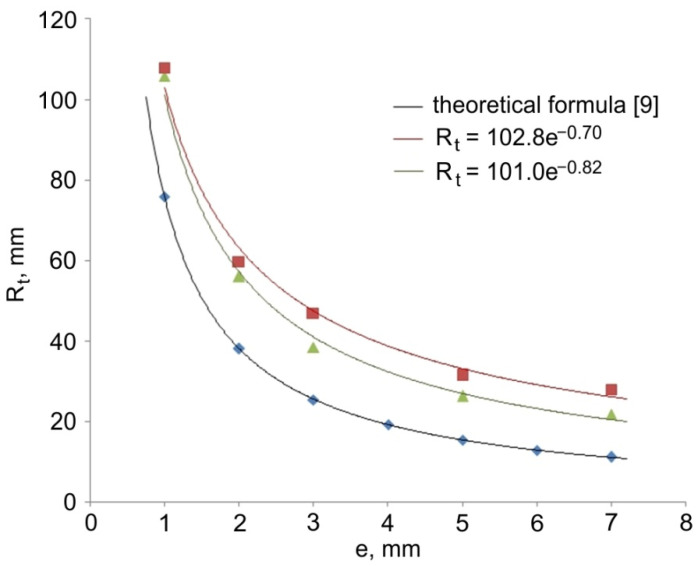
Comparison of the theoretical and numerical modelling values of torus radius R_t_ (outer radius of workpiece 2R = 20 mm, inner radius of workpiece 2r = 18 mm, diameter of stock 2R_k_ = 40 mm, friction coefficient 0.2 and 0.5).

Table 4 shows the values of the radius R_t_ of the torus with an outer diameter of 20 mm and an inner diameter of 18 mm, obtained in the numerical modelling after forming the extruded pieces from a stock with a diameter of 40 mm. Numerical modelling was carried out for friction coefficients of 0.2 and 0.5. The data in Table 4 were compared with the values calculated on the basis of the theoretical formula [9]. The trend of changes in the radius of torus is in good agreement with the values obtained by the finite element method. However, numerical results overestimated the values of the torus radius (Figure 17).

With respect to the scale of residual stress, this has been divided into three categories [43,44]: Type I, where σ_I_ refers to macro-scale stress which varies within the material over a length-scale much larger than the grain size. Type II and Type III are meso- and micro-scale residual stress. Lead has a recrystallization temperature below room temperature [36,37]. Thus, lead does not harden at room temperature. In the temperature of lead processing, stress-free grains appear in the structure.

The stability of the plastic flow of lead during the formation of a hollow torus extruded piece is only possible in the case of using proper tool shapes, providing the possibility of forming in the presence of all-side compressive stresses. It has been found, based on the analysis of eccentric extrusion simulation results, that these conditions are satisfied by the eccentric die, which forces the metal flow with a contribution of a sufficiently high mean compressive stress. Based on the simulation results, it has been determined that the course of metal deformation is controlled by the die walls, bar, and the ram.

## 7. Conclusions

The proposed eccentric direct extrusion process is useful in the case of making thin-walled components from materials that cannot be bent. The results of experimental investigations and numerical modelling allow the following conclusions to be drawn:

- The obtained die stampings have uniform wall thicknesses and cross-sectional shapes around their circumference. They are characterised by good surface quality and favourable mechanical properties resulting from forming without damaging the metal structure. It ensures better operational properties of the elements obtained;
- The investigated technological process of forming the hollow toric elements makes it possible to obtain the desired arbitrary curvature of the torus axis in one operation, and does not require additional technological finishing;
- The value of the radius R_t_ of the elements formed by the direct extrusion method from a specific stock is inversely proportional to the value of the eccentric e. The height of the material does not directly affect the radius of the torus Rt. The proposed method of extrusion uses the phenomenon of a uniform flow of material with a different volume in relation to the symmetry plane of the calibrating pin in the die;
- In the case of using a die with a conical bottom with an angle close to the stabilization zone of the material flow, the extrusion force decreases. The derived geometrical relationships shows that the angle Θ of the cone of die bottom has no direct impact on the radius R_t_ of the torus curvature;
- By slide out of the calibrating mandrel below the plane of the die bottom, it is possible to experimentally increase the radius R_t_ of the extruded piece in relation to the analytically determined value of this radius;
- Along with the decreasing coefficient of friction between the extruded material and the die, the radius of the curvature of the extruded part Rt increases in relation to the calculated theoretical formula [9];
- A significant increase in the eccentric displacement of the calibration gap in the die may affect the proper flow of the workpiece material;
- The proposed design of the tooling for direct extrusion with an eccentric die opening shows a satisfactory durability, can be easily adapted in order to obtain a large range of dimensions of extruded elements, and enables simple operation.

## Figures and Tables

**Table 1:** Basic mechanical properties of Pb1 lead.

Yield Stress R_e_, MPa	Ultimate Tensile Stress R_m_, MPa	Elongation A_5_, %	Young’s Modulus E, MPa	Poisson’s Ratio ν
5	20	50	0.16×10^5^	0.44

**Table 2:** Torus radius R_t_ obtained experimentally for different values of eccentricity e (three test series, outer radius of workpiece 2R = 20, inner radius of workpiece 2r = 18 mm, wall thickness 1 mm).

Eccentric e, mm	Number of Experiment	Radius of the Toric Part of the Pipe R_t_, mm
1	1	78.7
	2	76.8
	3	79.2
2	1	40.1
	2	39.1
	3	42.7
3	1	27.2
	2	27.5
	3	30.0
5	1	20.1
	2	17.3
	3	22.1
7	1	14.7
	2	13.3
	3	15.9

**Table 3:** Torus radius R_t_ obtained experimentally for different values of eccentricity e (three test series, outer radius of workpiece 2R = 22, inner radius of workpiece 2r = 18 mm, wall thickness 2 mm).

Eccentric e, mm	Number of Experiment	Radius of the Toric Part of the Pipe R_t_, mm
1	1	85.4
	2	84.5
	3	79.6
2	1	37.6
	2	42.0
	3	38.7
3	1	25.4
	2	31.9
	3	25.5
5	1	15.8
	2	18.9
	3	15.3
7	1	11.1
	2	12.6
	3	10.2

**Table 4:** Torus radius R_t_ obtained in numerical modelling for different values of eccentricity e (outer radius of workpiece 2R = 20, inner radius of workpiece 2r = 18 mm, wall thickness 1 mm).

Eccentric e, mm	Radius of the Toric Part of the Pipe Rt, mm (Friction Coefficient 0.2)	Radius of the Toric Part of the Pipe Rt, mm (Friction Coefficient 0.5)
1	107.9	105.8
2	59.7	55.9
3	46.9	38.3
5	31.3	26.2
7	27.8	21.7

## Data Availability

The data presented in this study are available on request from the corresponding author.
